# Dexmedetomidine decreases the emergence agitation in infant patients undergoing cleft palate repair surgery after general anesthesia

**DOI:** 10.1186/s12871-015-0124-7

**Published:** 2015-10-13

**Authors:** Wei Peng, TieJun Zhang

**Affiliations:** Department of Anesthesiology, School and Hospital of Stomatology, Wuhan University, No. 237 Luoyu Street, Wuhan, Hubei 430079 China

**Keywords:** Dexmedetomidine, Emergence agitation, Cleft palate repair surgery, Sevoflurane, General anesthesia

## Abstract

**Background:**

To determine whether continuous intravenous infusion of dexmedetomidine (DEX) can affect the incidence of Emergence Agitation (EA) after general anesthesia in infant undergoing cleft palate repair surgery.

**Methods:**

Forty infants underwent cleft palate repair surgery under general anesthesia were randomly divided into the DEX (D) group and Placebo (P) groups. Patients in group D received continuous intravenous infusion of DEX 0.8 μg · kg-1 · min-1 after the induction. Patients in group P were administered with continuous intravenous infusion of the equivalent volume of normal saline. Both groups were induced with fentanyl 0.005 mg/Kg, propofol 2 mg/Kg and cisatracurium 0.2 mg/Kg. Anaesthesia was maintained with continuous intravenous infusion of propofol (2 mg/Kg · h), remifentanil (0.1 μg/Kg · h), and inhalation of 1 to 3 % sevoflurane.

**Result:**

The heart rate (HR) in group P was significant higher than that in group D at the time of operation (*P* < 0.05), postoperative 15 min, 30 min and the time of extubation (*P* < 0.01). The mean arterial pressure (MAP) in group P was higher comparing with MAP in group D at the time of extubation (*P* < 0.05). The spontaneous eye opening times and spontaneous arm or leg motion times were longer in group D (*P* < 0.05). The mean agitation scores of patients in group D were significantly lower than that in group P (*P* < 0.01). However, the incidence of EA in group P and group D was 90 % and 15 % (*P* <0.05).

**Conclusion:**

The continuous intravenous infusion of DEX after induction could significantly reduce the occurrence of EA.

**Trial registration:**

The Chinese Clinical Trial Register ChiCTR-TRC-13003865

## Background

Cleft lip and palate as the most common craniofacial abnormalities in paediatric pathology had an incidence in 1.8 % in China [1]. Cleft palate repair is one of the most common oral and maxillofacial sugery in children and it may be associated with significant postoperative pain. A minority of infants aged < 3 years could tolerate the surgery without general anesthesia. However, anesthetic management during the cleft palate surgical repair always has a high rate (13 %) of postoperative complications including postoperative swelling of the tongue, bleeding which maybe because the surgical procedure and the complications also included pain, nausea and vomiting, wound dehiscence, bronchospasm, emergence agitation or delirium, which may be ascribed to the choice of anesthesia [2, 3]. Emergence agitation (EA) combined with vigorous crying may lead to wound dehiscence and pulmonary complications that might result in delayed recovery and a prolonged hospital stay. Much effort has been made to avoid these complications, and to improve the quality of anesthesia, however, there is no consensus on the safest anesthetic agents for pediatric patients undergoing surgical repair of cleft palate.

The typical anesthesia method of infant is inhalational anesthetic. Sevoflurane, as a popular inhalational anesthetic for children, has been routinely used because it is less pungent and has a more rapid onset and offset because of lower solubility in blood, fast recovery properity, relative lack of airway irritation and greater hemodynamic stability compared with other inhaled agents [4, 5]. However, studies had showed that sevoflurane had a relatively high incidence of EA in infants [6, 7] even in the absence of any surgical intervention [8]. EA in pediatric patients is a clinical entity generally defined by behaviors including combativeness, excitation, disorientation and inconsolability [9]. The incidence of EA is widely ranging in the literature from 10–80 % and it is usually a self-limited phenomenon, but can be severe and present dangers to both patients and caregivers [9]. EA has a phenomenon of nonpurposeful restlessness and agitation, thrashing, crying or moaning, disorientation, and incoherence [9], which frequently happened when children recovering from anesthesia and can create a challenging situation to their health care providers.

DEX is a highly specific, potent and selective α2-adrenoceptor agonist, which has sedative, anxiolytic and analgesic properties. It has a α2/α1 selectivity ratio of 1600:1, which is eight times more effective compared with clonidine [10]. DEX has been used effectively in intensive care to aid weaning from mechanical ventilation [11] and now is being utilized increasingly in infant anaesthetic [12–14]. It was reported that DEX can eliminate pain as a potential source of discomfort and agitation [15, 16].

In this study, we aimed to explore whether the prophylactic use of DEX could reduce the incidence of EA in infants undergoing cleft palate repair and confirm the edative, anxiolytic and analgesic effects of DEX in clinical treatment of cleft lip and palate.

## Methods

The study was approved by the institutional ethics committee of the School and Hospital of Stomatology of Wuhan University (Protocol 2013–46, Date: March 1, 2013) and registered with the Chinese Clinical Trial Register (ChiCTR -TRC-13003865).

### Patients and study design

This clinical trial was reviewed and approved by the Ethics Committee of the School and Hospital of Stomatology of Wuhan University. This study was conducted at School and Hospital of Stomatology of Wuhan University in accordance with the declaration of Helsinki. The written informed consent was obtained from parents of each infant patient in our study. This study enrolled 40 American Society of Anesthesiologists (ASA) physical status I patients aged from 3 months to 24 months scheduled to undergo cleft palate repair. Exclusion criteria included bradycardia, influenza, coagulopathy or major systemic illness. All operations were performed by the same experienced surgeon. The enrolled patients were randomly divided into group D (continuous infusion 0.8 μg · kg-1 · min-1 dexmedetomidine) and group P (continuous infusion the same as the volume of IV normal saline) sing a computer-generated sequence of numbers and a sealed envelope assignment which were prepared and kept by a research coordinator.

### Surgical procedure and clinical observations

Vital signs were monitored and recorded throughout the study. Standard monitoring included electrocardiograph (ECG), blood pressure and peripheral oxygen saturation. Basal anaesthesia was administrated by the inhalation of 8 % sevoflurane in 100 % oxygen via a face mask with spontaneous ventilation. Patients in group D and group P were induced with fentanyl 0.005 mg/Kg, propofol 2 mg/Kg and cisatracurium 0.2 mg/Kg. Anaesthesia was maintained with continuous infusion of propofol (2 mg · kg-1 · h-1), remifentanil (0.1 μg · kg-1 · h-1) and inhalation of 1 to 3 % sevoflurane. Before the end of surgery, analgesia pump was connected. Analgesia formula: total fentanyl 20 μg · kg-1 was diluted to 100 ml 0.9 % saline with 0.4 μg · kg-1 · h-1 constant rate infusion.

Oral endotracheal intubation of appropriate size for the age and weight of the child were placed after the induction. Patients in group P and group D respectively received continuous intravenous infusion of saline and dexmedetomidine 0.8 μg · kg-1 · min-1 after the induction. At the end of surgery, the fresh gas flow was increased to 6 L · min-1, and the effect of the paralytic agent, cisatracurium, was reversed by neostigmine (0.04 mg/Kg). Atropine (0.01 mg/Kg) was co-administrated with neostigmine. The trachea was extubated on resumption of spontaneus respiration and control of airway. All the patients received pain relief.

The heart rate (HR), mean arterial pressure (MAP), and SpO2 were recorded every 5 min from the time of induction in operation room. EA was assessed with 5-point scale (Table 1) [17] and scoring system for emergence agitation (behavior score): sleeping, 1 score; awake and calm, 2 score; irritable and crying, 3 score; inconsolable crying, 4 score; severe restlessness and disorientation, 5 score. EA was recorded every 5 min from the time of discontinuation of the anaesthetic until the patients were awake, alert, calm, and responsive to the parents. Anesthesia and procedure times and per operative and postoperative side effects were recorded.

**Table 1:** Five point scale

Scoring system for emergence agitation (behavior score)	
Sleeping	1
Awake and calm	2
Irritable and crying	3
Inconsolable crying	4
Severe restlessness and disorientation	5

### Statistics analysis

Before initiating the study, a power analysis suggested that a sample size of 20 patients in each group should be adequate to detect a 30 % reduction in extubation time and agitation score with a power of 0.8 and α of 0.05. The results were expressed as [mean ± SD, n (%)]. Student's *t* test was used in the comparisons of age, weight, operating, anesthesia, emergence agitation score, and recovery time. The gender, ASA and postoperative side effects in two groups were compared using *chi*-square and Fisher's exact test. Bonferroni adjustment was used in the comparisons of intragroup values of MAP, HR, and SpO2.

## Results

**Fig. 1:**
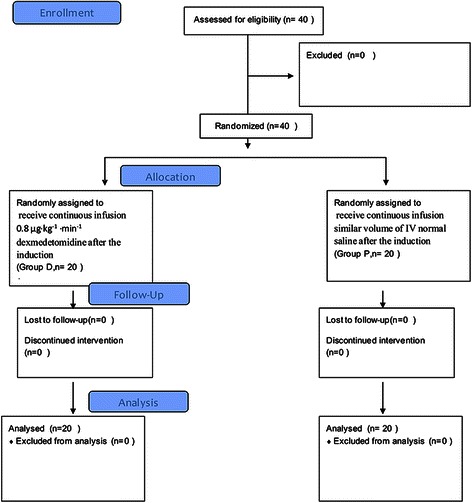
Consort flow diagram

A total number of forty infant patients were enrolled in this study (20 in Group D and 20 in Group P). All the patients participated in the study were followed up for the observational period and the flow chart of this study was shown in Fig. 1. There were no significant differences in gender, age, body weight, ASA, during of operation and during of anesthesia between two groups (Table 2).

**Table 2:** Demographic and anesthesia data

	Group D (*n* = 20)	Group P (*n* = 20)
Age (years)	12.5 ± 3.8	11.3 ± 4.5
Gender (M/F)	13/7	14/6
Weight (Kg)	9.6 ± 1.9	9.1 ± 1.6
ASA (I/II)	15/5	16/4
During of operation (min)	70.6 ± 32.2	74.8 ± 33.2
During of anesthesia (min)	102.9 ± 34.0	116.8 ± 30.4

Values are mean ± SD or numbers. Differences not significant

**Fig. 2:**
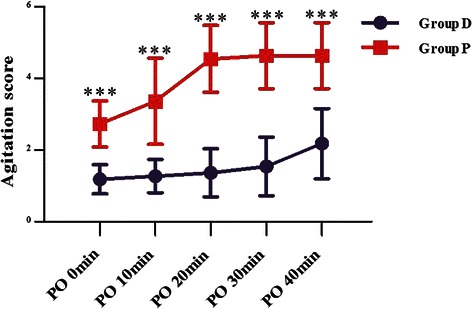
Heart rate between the two groups. Values are expressed as mean ± SD (min). *P < 0.05 and & *P* < 0.01 for group D vs P

**Fig. 3:**
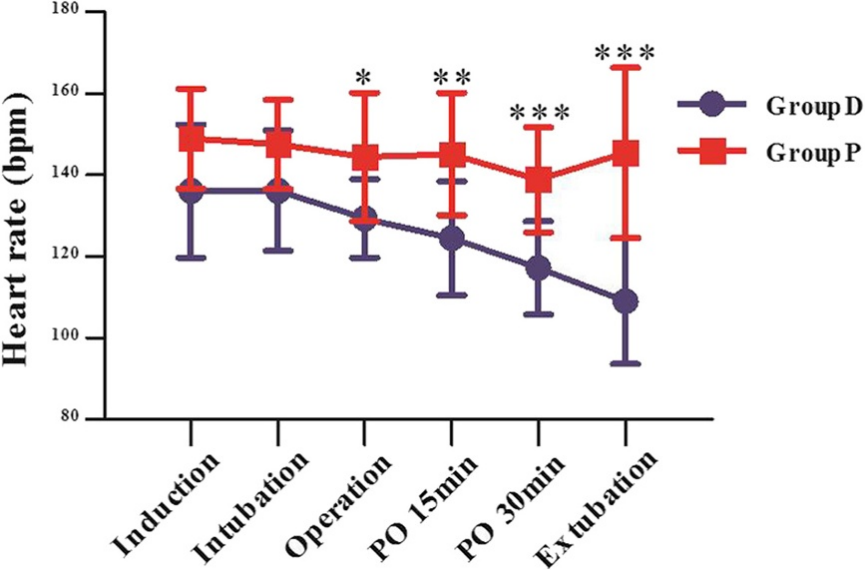
MAP between the two groups. Values are expressed as mean ± SD (min). *P < 0.05 for group D vs P

Comparing with group D, the HR was higher in group P during the time of operation (*P* = 0.014), postoperative 15 min (*P* = 0.003), postoperative 30 min (*P* = 0.0004) and extubation (*P* = 0.0001) (Fig. 2). The MAP in group P was higher than that of group D at extubation (*P* = 0.027). The SpO2 data were similar between two groups (Fig. 3). Intravenous injection with 0.25 mg/kg Methoxamine Hydrochloride and 0.03 mg/kg Atropine were performed to tackle the hypotension and bradycardia, respectively.

The respiratory recovery time and remove extubation time were similar in the two groups. The spontaneous eye opening time and spontaneous arm or leg motion time was shorter in the group P than that in the group D (*P* = 0.027 and *P* < 0.0001). There was no significant difference in the time of discharge to the PACU between two groups (Table 3).

**Table 3:** Recovery parameters and the incidence of agitation was 90 % in group P and 15 % in group D

	Group D (*n* = 20)	Group P (*n* = 20)
Respire recovery time	4.3 ± 1.6	3.9 ± 1.6
Remove extubation time	7.8 ± 1.5	6.5 ± 1.4
Spontaneous eye opening time	33.2 ± 11.0*	17.8 ± 6.1
Spontaneous arm or leg motion time	25.6 ± 13.7*	9.1 ± 3.0
Discharge to recovery room	55.1 ± 3.0	54.5 ± 2.8
Bronchospasm	1	3
Nausea-vomiting	1	2
Emergence agitation	3 (15 %)	18* (90 %)

Values are expressed as mean ± SD (min). *P < 0.05 for group D vs P

**Fig. 4:**
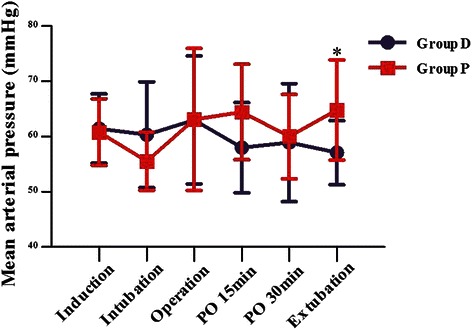
Agitation scores [mean (SD)]. Values are expressed as mean ± SD (min) & *P* < 0.0001 for group D vs P

The mean agitation scores in the dexmedetomidine group were significantly lower than the P group at PO 0 min, 10 mim, 20 min, 30 min and 40 min & (*P* < 0.0001) (Table 3) (Fig. 4).

## Discussion

Cleft lip and palate is the most common congenital facial malformation in humans. It occurs with ethnic and geographic variation [17]. This disorder is generally divided into two groups: clefts involving the lip with or without cleft palate and isolated cleft palate [2]. Cleft lip and palate can also be part of a syndrome when it is associated with other congenital defects.

Many factors can contribute to the high rate of EA after sevoflurane anesthesia in infant patients undergoing cleft palate repair surgery and these factors included surgical procedure, skill of anesthesia management, separating from the family, incapacity and loss of independence. Cleft palate repair surgery has specific complications associated with the surgical procedure [2], such as postoperative laryngeal edema, Specific complications such as postoperative laryngeal edema may associated with the surgical procedure which occurred during the cleft palate repair surgery and it can result in impeded breathing and blocking of breathing system. In addition, the repair of cleft palate leads to significant pain in the postoperative period. Inadequate pain relief may be the cause of agitation, particularly after short surgical procedures for which peak effects of analgesics may be delayed until the child is completely awake [18]. Since postoperative pain is considered to be one of the major causes of EA, therefore, it is generally believed that reducing or eliminating pain may decrease the incidence of EA. It has been reported in several previous studies that regional block, opioids, and nonsteroidal antiflamatory drugs could decrease the incidence of EA [19–22]. Prophylactic propofol appears to be effective for reducing the incidence and severity of EA in children emerging from general anesthesia [23]. Transition to propofol at the end of sevoflurane anesthesia reduces the incidence of EA and improves the quality of emergence. There is a small increase in recovery time, but no delay in discharge home [24].

Although pain may be the cause of postoperative agitation after general anesthesia, however, there were still other factors which may induce the occurrence of EA [25]. And some studies suggest that EA can be provoked without pain. It had been reported in the study of Isik et al. [26] that the incidence rate of EA was 48 % in pediatric patients under sevoflurane anesthesia when undergoing magnetic resonance imaging. In our study, patients in both groups received similar pain relief, however, there was a higher incidence of EA in group P comparing with patients in group D.

Sevoflurane is related to the high incidence of EA, and there is a common agreement amongst anesthetists that sevoflurane can increase the incidence of EA in the postoperative stage in pediatric patients compared to propofol or halothane. Meta-analysis demonstrated that EA happened more frequently in children under sevoflurane anesthesia than propofol anesthesia [27]. Kuratani and Oi [28] showed that there was a high rate of EA in pediatric patients with sevoflurane anesthesia compared with patients with halothane. A higher incidence of EA was also recorded in patients who received sevoflurane for nopainful interventions, such as eye examinations [29]. The reasons for a higher incidence of EA after sevoflurane were not fully understood. Using the electroencephalography during sevoflurane anesthesia, it has been found that epileptiform seizure activity was observed in non-epileptic patients and sevoflurane was believed to have a specific side effect on the central nervous system [30, 31].

DEX, a selective α-2 adrenoceptor agonist with sedative, analgesic, and anxiolytic propery without significant resporatory depression at clinical dosages, has been widely used in pediatric and adult populations [32–35]. Guler reported that DEX given 5 min before the end of surgery, was effective in reducing EA with prolongation of extubation and emergence times in children undergoing adenotonsillectomy [36]. Ibacache et al. reported that children undergoing lower abdominal and genital surgery from 1 to 10 years anesthetized 1–3 % sevoflurane in 50–50 % O2/N2O and a single i.v. dose of DEX after induction, resulted in a reduction of postoperative agitation from 37 % in the placebo group to 17 and 10 % with 0.15 and 0.3 μg · Kg-1, respectively. The results of this study indicated that continuous intravenous infusion of DEX 0.8 μg · kg-1 · min-1, after anesthesia induction, reduced the incidence of emergence agitation following sevoflurane anesthesia in infant patients undergoing cleft palate repair surgery.

In our studies, all infants were pre-induced with 8 % sevoflurane until the venous access was established. Anaesthesia was maintained with a continuous infusion of propofol (2 mg/Kg · h), remifentanil (0.1 μg/Kg · h), and inhalation of 1 to 3 % sevoflurane. Patients in group D received continuous intravenous infusion of DEX (0.8 μg · kg^−1^ · min^−1^). The HR of patients in group D was lower than group P after induction, however, it was still within a safe range. Besides, the lower HR could decrease the myocardial oxygen consumption. MAP in Group D was more stable than group P. The increasing HR and MAP of patients in group P in the extubation period may due to the reason of higher severe level of agitation and an decrease of HR and MAP of patients in group D during the extubation period may be attributed to the effect that dexmedetomidine reduces plasma norepinephrine levels [37].

We observed 90 % agitation in group P and 15 % in group D underwent cleft palate repair surgery under general anesthesia. The higher incidence of EA found in placebo group compared with the studies of Guler et al. and Ibacache et al., may be attributed to the cleft palate repair. Comparing with the results in the study of Guler et al. and Ibacache et al., the lower incidence of EA in group D may due to the higher dose of dexmedetodine. In our study, we found the fact that the infants administered dexmedetomidine could slightly prolong respire recovery time and remove extubation time. The spontaneous eye opening times and spontaneous arm or leg motion times was obviously longer in group D (*P* < 0.0001), which showed the sedative effects dexmedetomidine. The time to discharge to PACU was similar in the two groups.

Sandner-Kıeslıng et al. [38] concluded that nausea and vomiting was seen in 30 % following sevoflurane anesthesia for magnetic resonance imaging. Two cases of nausea and three cases of bronchospasm were observed as side effects in group P, while one case of nausea and two cases of bronchospasm were observed as side effects in group D.

As a result, continuous infusion of 0.8 μg · kg-1 · min-1 DEX after induction could significantly decrease the occurrence of emergence agitation in infants undergoing cleft palate repair with sevoflurane anesthesia. The continuous infusion of DEX in this dose was safe and it could lead to a decrease incidence of side effects. The limitation of the current study was the samples size of the surgery cases of this research.

## Conclusions

We concluded that continuous intravenous infusion of DEX 0.8 μg · kg-1 · min-1 after induction significantly decreases the occurrence of EA in infants undergoing cleft palate repair under general anesthesia, which may be helpful for the further treatment.

## Abbreviations

DEX: Dexmedetomidine (DEX)
Group P: Group Placebo
HR: Heart rate
MAP: Mean arterial pressure
EA: Emergence Agitation
ASA: Anesthesiologists
